# Application of AI-assisted MRI for the identification of surgical target areas in pediatric hip and periarticular infections

**DOI:** 10.1186/s12891-024-07548-1

**Published:** 2024-06-01

**Authors:** Yuwen Liu, Lingyu Chen, Mingjie Fan, Tao Zhang, Jie Chen, Xiaohui Li, Yunhao Lv, Pengfei Zheng, Fang Chen, Guixin Sun

**Affiliations:** 1 Department of Orthopaedic Surgery, Children’s Hospital of Nanjing Medical University, Nanjing, China; 2 Department of Computer Science and Engineering, Nanjing University of Aeronautics and Astronautics, Nanjing, China; 3 Department of Orthopaedic Surgery, Qinghai Women’s and Children’s Hospital, Xining, China; 4 Department of Orthopaedic Surgery, Wuxi Children’s Hospital, Wuxi, China; 5 Department of Radiology, Children’s Hospital of Nanjing Medical University, Nanjing, China; 6 Department of Traumatic Surgery, Shanghai East Hospital, Nanjing Medical University, Shanghai, China

**Keywords:** Artificial intelligence, MRI, Children, Hip, Infection

## Abstract

**Objective:**

To develop an AI-assisted MRI model to identify surgical target areas in pediatric hip and periarticular infections.

**Methods:**

A retrospective study was conducted on the pediatric patients with hip and periarticular infections who underwent Magnetic Resonance Imaging(MRI)examinations from January 2010 to January 2023 in three hospitals in China. A total of 7970 axial Short Tau Inversion Recovery (STIR) images were selected, and the corresponding regions of osteomyelitis (label 1) and abscess (label 2) were labeled using the Labelme software. The images were randomly divided into training group, validation group, and test group at a ratio of 7:2:1. A Mask R-CNN model was constructed and optimized, and the performance of identifying label 1 and label 2 was evaluated using receiver operating characteristic (ROC) curves. Calculation of the average time it took for the model and specialists to process an image in the test group. Comparison of the accuracy of the model in the interpretation of MRI images with four orthopaedic surgeons, with statistical significance set at *P* < 0.05.

**Results:**

A total of 275 patients were enrolled, comprising 197 males and 78 females, with an average age of 7.10 ± 3.59 years, ranging from 0.00 to 14.00 years. The area under curve (AUC), accuracy, sensitivity, specificity, precision, and F1 score for the model to identify label 1 were 0.810, 0.976, 0.995, 0.969, 0.922, and 0.957, respectively. The AUC, accuracy, sensitivity, specificity, precision, and F1 score for the model to identify label 2 were 0.890, 0.957, 0.969, 0.915, 0.976, and 0.972, respectively. The model demonstrated a significant speed advantage, taking only 0.2 s to process an image compared to average 10 s required by the specialists. The model identified osteomyelitis with an accuracy of 0.976 and abscess with an accuracy of 0.957, both statistically better than the four orthopaedic surgeons, *P* < 0.05.

**Conclusion:**

The Mask R-CNN model is reliable for identifying surgical target areas in pediatric hip and periarticular infections, offering a more convenient and rapid option. It can assist unexperienced physicians in pre-treatment assessments, reducing the risk of missed and misdiagnosis.

## Introduction

Given the various pathogenic factors, the diversity of affected regions, and the unpredictable sequelae and complications, pediatric musculoskeletal infections have remained a focal topic of research. The hip stands out as a primary site for septic arthritis in children [1–5]. Compared to other joints, the harm and consequences it entails are more severe, including avascular necrosis of the femoral head, chondrolysis, leg length discrepancy, hip joint dislocation or subluxation, and growth retardation [6]. The etiology of hip infections in children can be attributed to bacterial colonization of the synovial membrane via a hematogenous route or can result from the adjacent osteomyelitis. In some cases, septic arthritis can be associated with psoas abscess causing hip symptoms [7]. Therefore, periarticular infections of the hip in children may not only manifest as joint abscesses but may also encompass adjacent infections, such as osteomyelitis, subperiosteal abscesses, and intramuscular abscesses [8–11]. Simple debridement and drainage of joints tends to neglect the management of adjacent infections, which would lead to prolonged hospitalization, increased costs, increased risk of reoperation, and a higher incidence of sequelae [2, 5, 10, 12–16].

MRI exhibits high sensitivity in the early diagnosis of musculoskeletal infections in children and can precisely show the extent of adjacent infections [17–19]. However, MRI sequences are numerous, and each image contains complex information as the infection progresses to the subacute or chronic stage. This complexity poses a challenge to unexperienced physicians in making a diagnosis. Accurate identification of osteomyelitis and abscess through MRI not only aids in localizing puncture sites for definitive diagnosis, but also serves as a critical factor in ensuring thorough debridement and drainage. This highlights the importance of using advanced imaging techniques for accurate and comprehensive diagnosis and treatment planning in pediatric hip infections.

Artificial intelligence(AI) holds significant potential in analyzing medical images. Through deep learning algorithms, AI can automatically detect and analyze abnormalities in images, assisting doctors in swiftly identifying lesions. Mask R-CNN [20] is a further improved network model developed based on the Faster R-CNN [21] framework, representing an advanced target detection algorithm in the field of AI. This model exhibits the capability to not only detect target regions within an image but also classify them based on the detected features, which closely resembles the diagnostic mindset of doctors. To the best of our knowledge, there have been no studies of AI-assisted MRI diagnostic models applied to pediatric periarticular infections of the hip. Thus in this study, we aim to construct an AI-assisted MRI model based on Mask R-CNN, and to investigate the feasibility in identifying surgical target areas in pediatric hip and periarticular infections.

## Materials and methods

### Study population

A retrospective study was conducted on 359 cases of hip and periarticular infections treated in three hospitals in China from January 2010 to January 2023. Among these cases, complete data and MRI examinations performed in 275 cases, aging of ≤ 14.00 years with an average age of 7.10 ± 3.59 years. Of these cases, 197 were male, and 78 were female. There were 166 cases of septic arthritis, 13 cases of acetabular osteomyelitis, 12 cases of proximal femoral osteomyelitis, 28 cases of septic arthritis combined with acetabular osteomyelitis, 32 cases of septic arthritis combined with proximal femoral osteomyelitis, 16 cases of septic arthritis combined with acetabular and proximal femoral osteomyelitis, and 8 cases of isolated soft-tissue infections alone.

The patients were included in the study based on the following criteria: (1) age of ≤ 14.00years; (2) hip and periarticular infectionsdefined as follows: (a) purulent fluid observed during percutaneous puncture, with positive puncture fluid or blood cultures; (b) purulent fluid observed during percutaneous puncture, without positive puncture fluid or blood cultures, while in combination with clinical history, physical examination, laboratory tests, and radiological findings indicative of infections; (c) postoperative pathology confirming infections. The exclusion criteria were: (1) autoimmune inflammatory diseases; (2) bone tumors or tumor-like lesions; and (3) poor imaging quality for clear visualization. This study obtained ethical approval from the ethics committees of Nanjing Children’s Hospital (Ethical Approval Number: 202301026-1), Wuxi Children’s Hospital (Ethical Approval Number: WXCH2023-02-029), and Qinghai Women’s and Children’s Hospital (Ethical Approval Number: 2022QHFELL 2KY).

### Establishment of MRI database

Of the 275 cases, 64 patients underwent repeat MRI examinations, resulting in a total of 339 MRI examinations performed. The axial STIR images were selected as the target training dataset, comprising a total of 7970 images, which were randomly divided into three groups: training group(5579 images), validation group(1594 images), and testing group(797 images) using a 7:2:1 split ratio. MRI scans were conducted using a 3.0T HDX MR scanner with a 16-channel phased-array coil or a 1.5T MR scanner with an 8-channel phased-array coil.

### Annotation of images

**Fig. 1:**
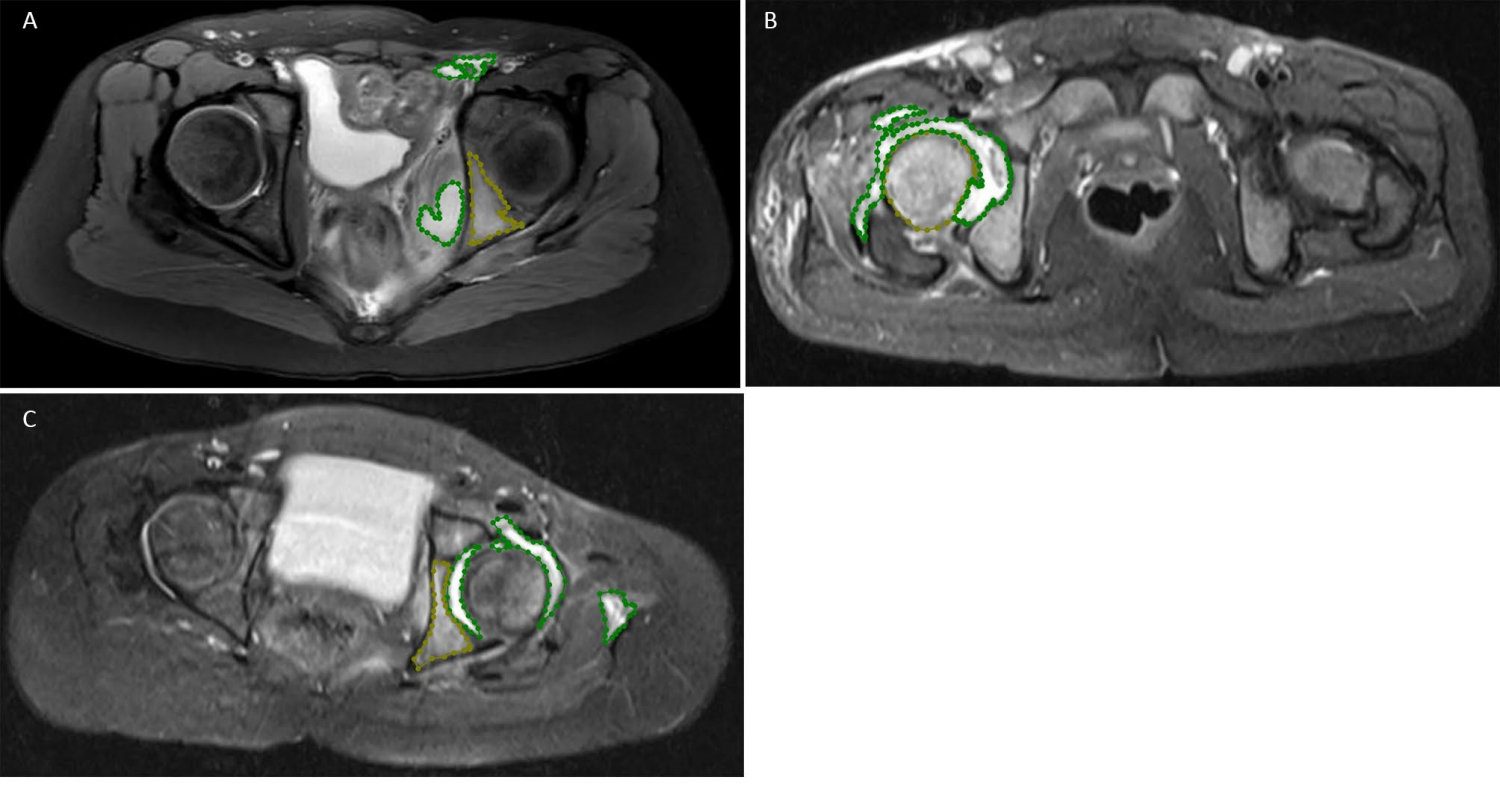
Labeling of hip joint axial STIR images using Labelme software. Bone marrow edema was labeled as label1 (yellow area) and the abscess as label2 (green area). (**A**) Left acetabular osteomyelitis with pelvic abscess. (**B**) Right hip septic arthritis with femoral osteomyelitis. (**C**) Left hip septic arthritis with acetabular osteomyelitis

The determination of the osteomyelitis and abscess involved the examination of coronal and axial STIR images of the hip. Regions with bone marrow edema and damage were considered as osteomyelitis [22]. This process was jointly completed by an orthopaedic specialist and a radiologic specialist with more than 15 years of experience. In case of disagreement, we consulted the pediatric chief radiologist who specializes in pediatric musculoskeletal infection imaging. For the axial STIR images, the Labelme software (http://labelme.csail.mit.edu) was used to outline the area of osteomyelitis marked as ‘label1’and the area of abscess marked as ‘label2’ (Fig. 1).

### Network framework

**Fig. 2:**
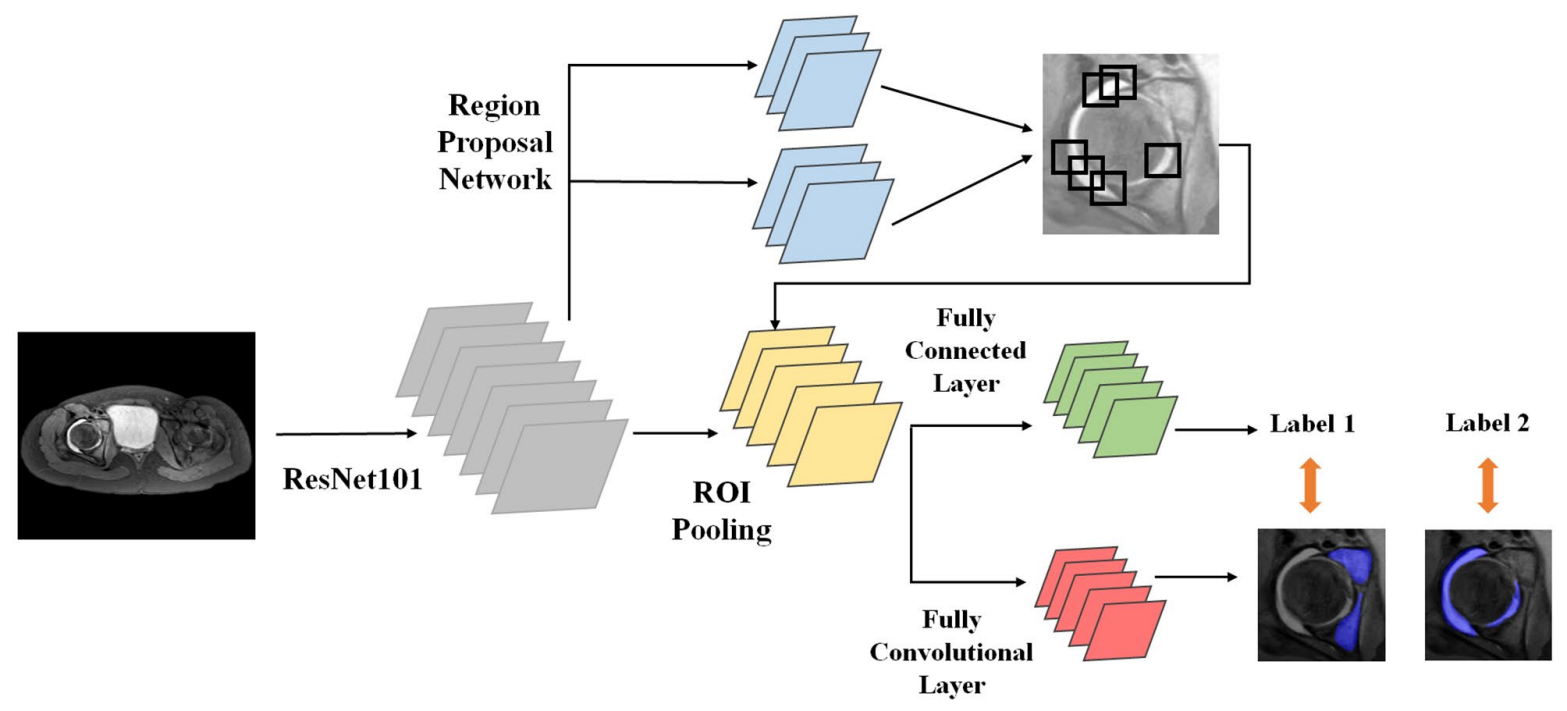
The framework used by the network

The collected image data was fed into a network based on the Mask R-CNN framework, which simultaneously performed classification and segmentation tasks. The detailed processing pipeline was as follows (Fig. 2). First, we chose the ResNet-101 architecture [23], which contains multiple convolutional layers, as the basic framework for extracting deep semantic and high-dimensional features containing spatial information. Next, the Region Proposal Network (RPN) was employed to process the obtained feature images and generate the corresponding bounding boxes of candidate objects that may contain important information. The model then combined the Regions of Interest (ROI) and feature images to predict the categories of osteomyelitis (label 1) and abscess (label 2) as well as the precise segmentation region by two separate convolutional branches.

Mask R-CNN is an improved network model based on the Faster R-CNN framework. The Faster R-CNN consists of two stages. The first stage introduces the RPN, consisting of a Fully Convolutional Neural Network (FCNN), to propose the bounding boxes of candidate objects. In this study, the model was configured to extract 2048-dimensional feature images to generate local neighbourhood regions, with each object corresponding to a score. The second stage is essentially Fast R-CNN [24], which involves pooling operations for ROIs, extracting feature vectors from the detected candidate boxes using Fully Connected Layers(FCL), and then performing the tasks of classification and bounding box regression. In addition to the class labels and bounding box offsets produced by Faster R-CNN, the Mask R-CNN adds a segmentation mask corresponding to the output of the third branch to the input samples, by proposing a binary mask for each ROI. The ResNet-101 was used as the convolutional backbone architecture for feature extraction over the whole image, achieving excellent gains in accuracy and speed. The network heads for bounding box recognition (classification and regression) and mask prediction for each ROI extended the network heads in Faster R-CNN into a more efficient and lightweight extended head. This head included the first five layers of the ResNet, suitable for computationally intensive data, enabling more precise segmentation mask results for label 1 and label 2.

### Training and validation of mask R-CNN

We employed a network model based on Mask R-CNN to achieve the recognition, classification, and segmentation of pediatric hip and periarticular infections. Based on previous research [21, 24–26], we iteratively optimized the Mask R-CNN network framework. The ResNet-101 network was used as the primary convolutional architecture to extract crucial features from MRI images. It is important to note that the foundational deep models of the ResNet series have already undergone training on the open-source ImageNet image database. These models could extract deep semantic features through down-sampling operations, such as the texture information of hip joints and lesion regions in MRI images. The network combined shallow and high-dimensional semantic features to achieve accurate classification and segmentation of MRI images.

In this study, the training group consisting of 5579 images was input into the Mask R-CNN network. Through convolutional and pooling layers, low-dimensional features were mapped into a high-dimensional space and further processed by FCL to obtain the output. After iterative training, the network gradually converged and stabilised.The validation groupof 1594 images was then fed into the trained Mask R-CNN network. The network performed feature extraction, candidate box extraction, pooling, and other steps to generate regression and classification results. These results included the categories and segmentation regions corresponding to osteomyelitis and abscess.

### Evaluation of mask R-CNN

The model was comprehensively analyzed using key metrics, including accuracy, sensitivity, specificity, precision, and F1 score. ROC curves were generated, and the AUC was calculated using the trapezoidal method. Calculation of the average time it took for the model and specialists to process an image in the test group.

### Comparison with diagnostic results from clinicians

Four orthopaedic surgeons were selected to participate in the interpreting of MRI images. Two of them had 2 years of experience (Doctor 1 and Doctor 2), while the other two had 5 years of experience (Doctor 3 and Doctor 4). The evaluation was performed on the images of the test group. To ensure fairness in the study, physicians who were involved in image collection, labeling, and model construction were excluded. All clinical information, including names, gender, age, hospital ID and dates, were concealed.

### Statistical analysis

Chi-square test was employed to compare the accuracy of the model and the four orthopaedic surgeons in identifyingosteomyelitis and abscess. All statistical analyses were conducted using SPSS 27.0 software (IBM Corp., Armonk, NY, USA), with statistical significance set at *P* < 0.05.

## Results

**Fig. 3:**
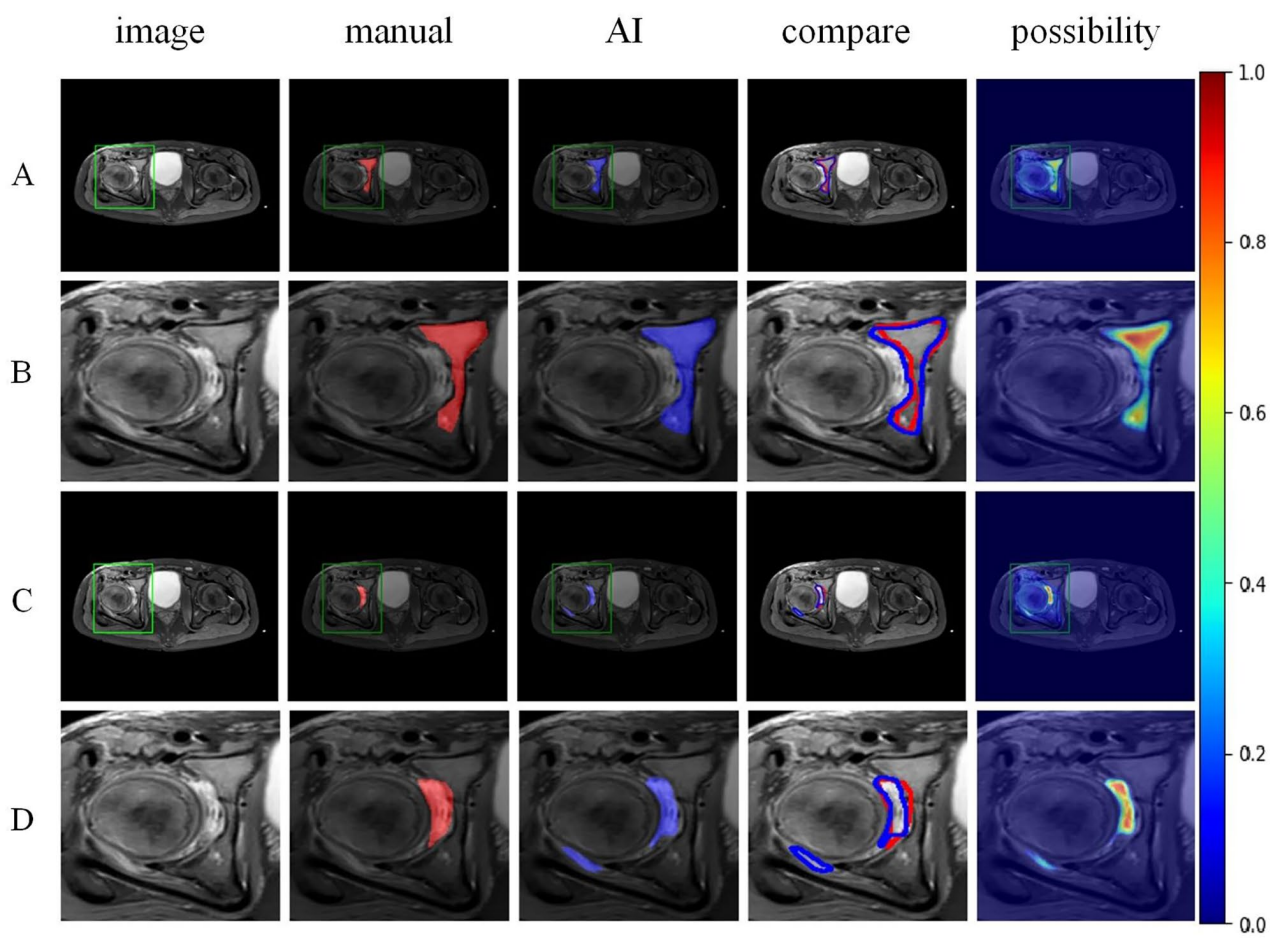
Axial STIR Images of pediatric hip infections. A series of axial STIR images of a pediatric hip were presented. The division of infected areas, including osteomyelitis and abscess, were labeled in red by the specialists. In contrast, the AI-generated diagnosis were depicted in blue, with the marker shade approaching crimson indicating a higher probability of infections

**Fig. 4:**
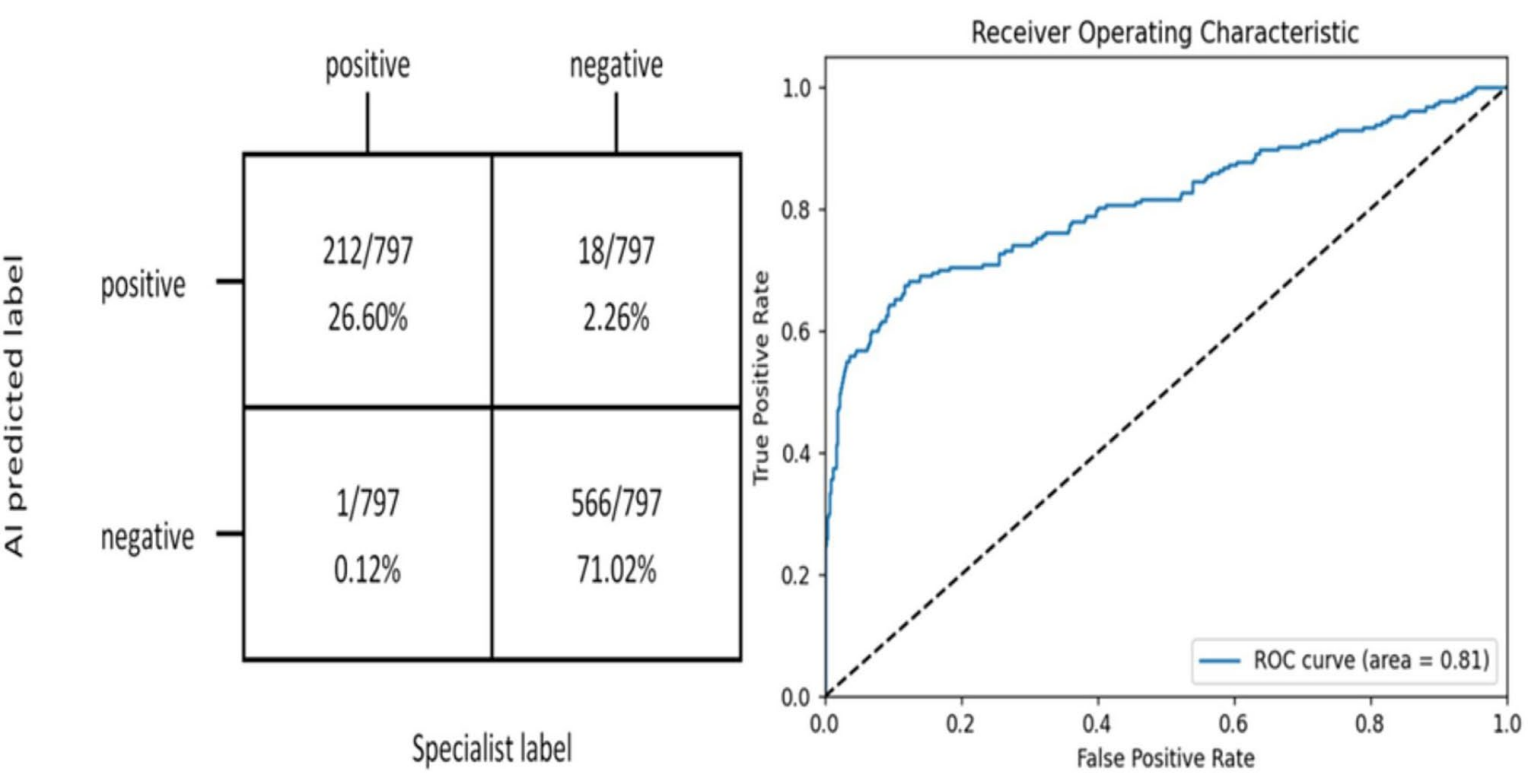
The matrix diagram and ROC curve reflected the effect of Mask R-CNN-based deep learning system on the identification of osteomyelitis

**Fig. 5:**
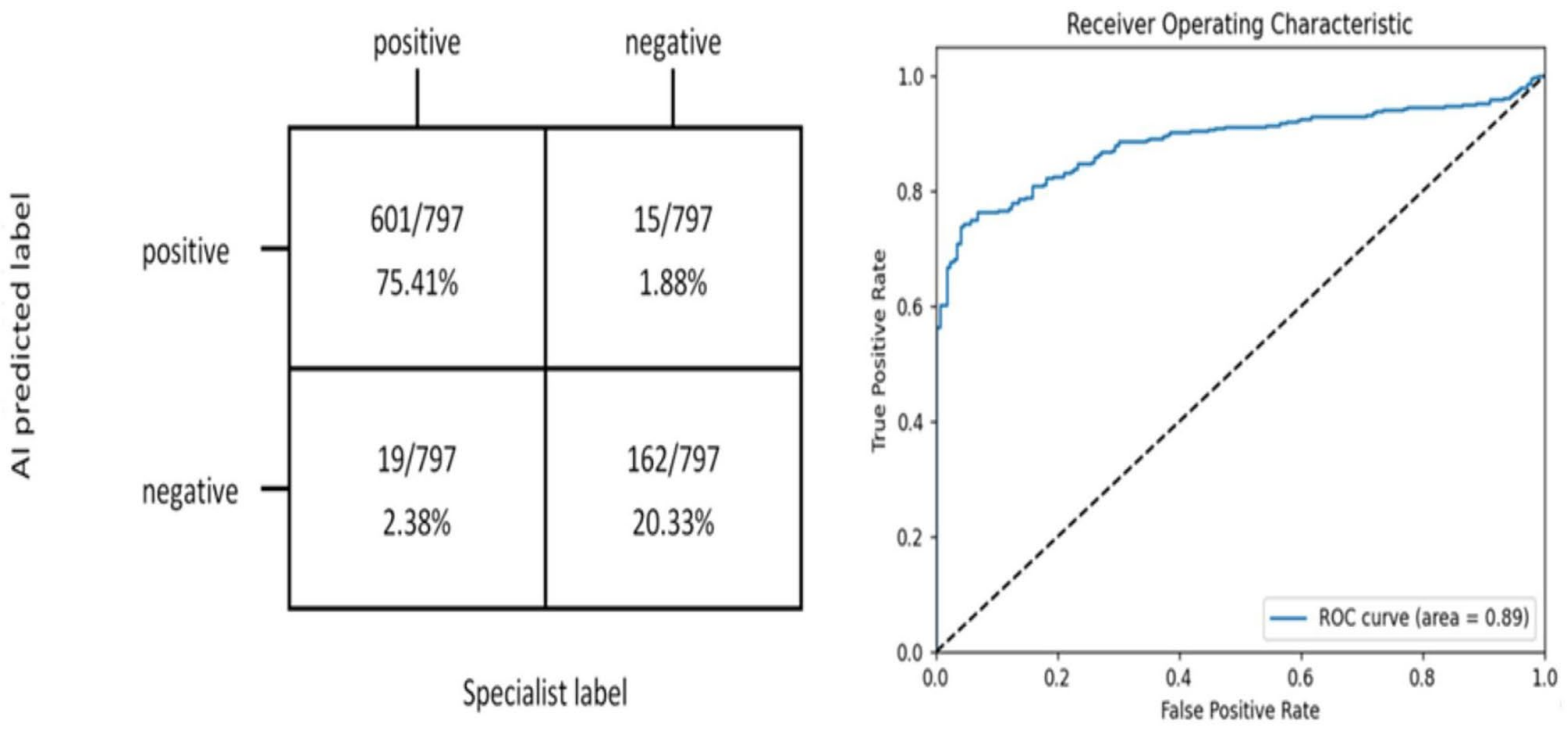
The matrix diagram and ROC curve reflected the effect of Mask R-CNN-based deep learning system on the identification of abscess

The Mask R-CNN model accurately identified and labelled the locations and risk probabilities of osteomyelitis and abscess in STIR images (Fig. 3). The performance of the model in identifying osteomyelitis and abscess in the test group and the ROC curves were shown in Figs. 4 and 5. The diagnostic performance metrics for label 1 were as follows: AUC of 0.810, accuracy of 0.976, sensitivity of 0.995, specificity of 0.969, precision of 0.922, and F1 score of 0.957 (Table 1). For label 2, the metrics were as follows: AUC of 0.890, accuracy of 0.957, sensitivity of 0.969, specificity of 0.915, precision of 0.976, and F1 score of 0.972 (Table 2). Additionally, the model demonstrated a significant speed advantage, taking only 0.2 s to process an image compared to average 10 s required by the specialists.

**Table 1:** Comparison of osteomyelitis identification between the Mask R-CNN model and orthopaedic surgeons

	Accuracy	*P*-value	Sensitivity	Specificity	Precision	F1 Score
Mask R-CNN	0.976		0.995	0.969	0.922	0.957
Doctor1	0.928	<0.001	0.883	0.945	0.855	0.868
Doctor2	0.925	<0.001	0.901	0.933	0.831	0.865
Doctor3	0.954	<0.001	0.944	0.957	0.889	0.916
Doctor4	0.937	<0.001	0.930	0.940	0.850	0.888

*P*-values were used to assess and compare the diagnostic accuracy between the Mask R-CNN model and doctors

**Table 2:** Comparison of abscess identificationbetween the Mask R-CNN model and orthopaedic surgeons

	Accuracy	*P*-value	Sensitivity	Specificity	Precision	F1 Score
Mask R-CNN	0.957		0.969	0.915	0.976	0.972
Doctor1	0.897	<0.001	0.924	0.802	0.942	0.933
Doctor2	0.887	<0.001	0.918	0.780	0.936	0.927
Doctor3	0.921	<0.001	0.944	0.842	0.954	0.949
Doctor4	0.912	<0.001	0.939	0.819	0.948	0.943

*P*-values were used to assess and compare the diagnostic accuracy between the Mask R-CNN model and doctors

**Fig. 6:**
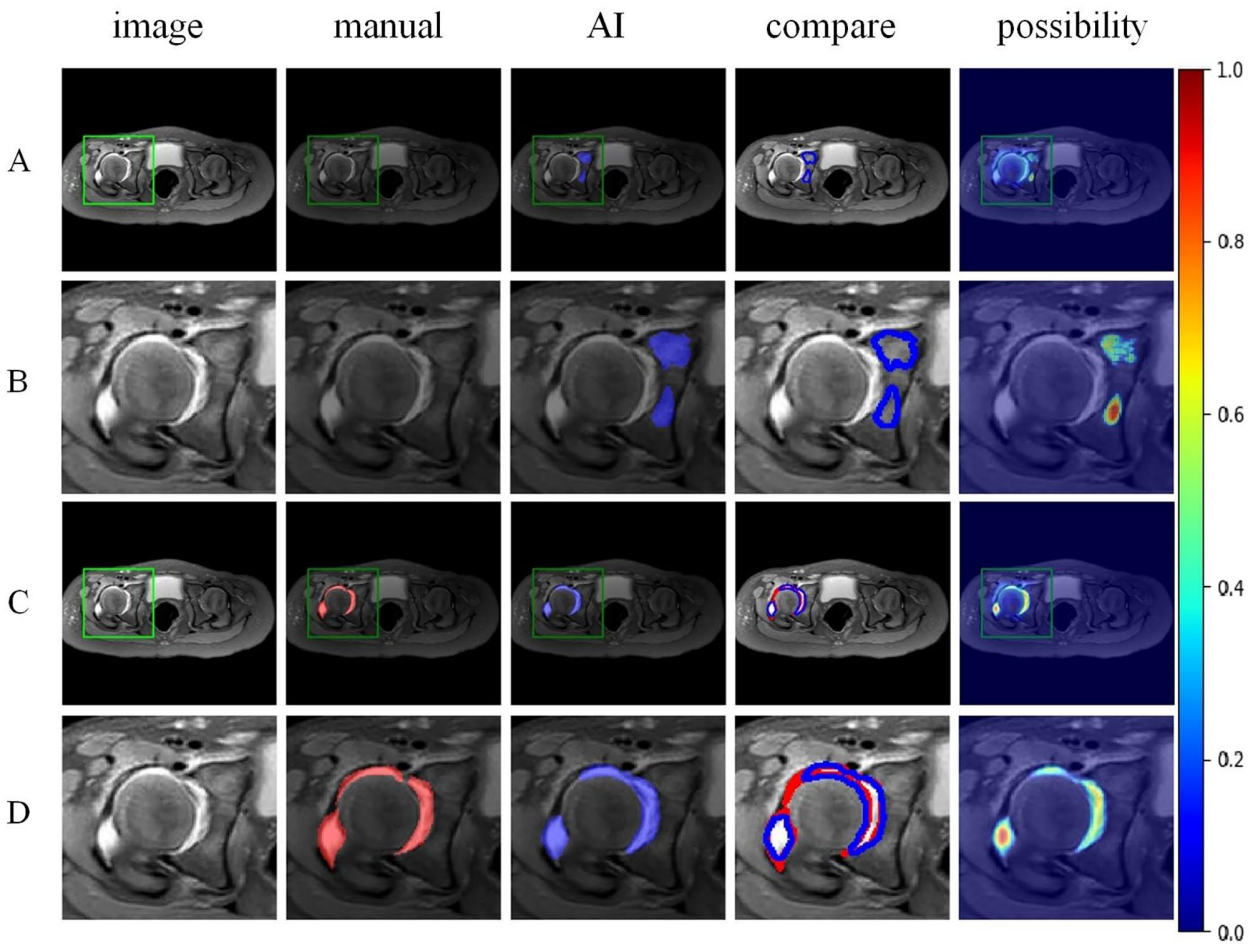
AI identified bone marrow edema in the right acetabulum which was not detected by the specialists (**Figure A** and **B**). AI agreed with expert diagnosis when identifying abscess (**Figure C** and **D**)

**Fig. 7:**
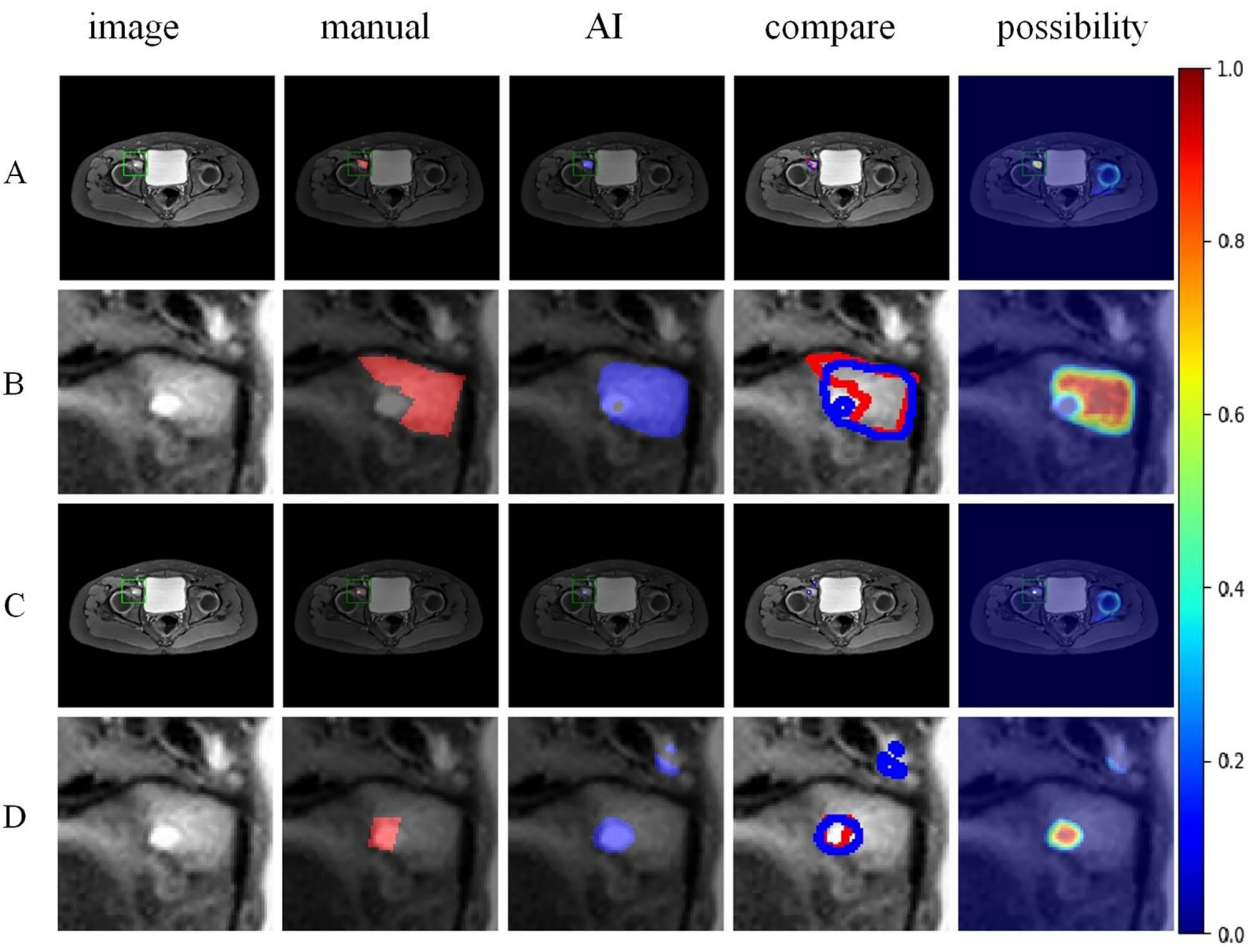
AI accurately identified small localised osteomyelitis (**Figure A** and **B**) and abscess (**Figure C** and **D**) at expert level

In the STIR images, osteomyelitis shows bone marrow edema with high signal and abscess shows fluid-like high signal. The model was more sensitive than the specialists to imaging changes in bone marrow edema (Fig. 6). And the model was equally reliable in identifying microinfections (Fig. 7).

The comparative performance of the model and the four orthopaedic surgeons for osteomyelitis diagnosis was shown in Table 1 and for abscess diagnosis was shown in Table 2. The accuracy for osteomyelitis diagnosis by the model (0.976) was significantly higher than that of the four orthopaedic surgeons (0.928, 0.925, 0.954, 0.937, respectively) with *P* < 0.05. Similarly, for abscess diagnosis, the model’s accuracy (0.957) was significantly higher than that of the four orthopaedic surgeons (0.897, 0.887, 0.921, 0.912, respectively) with *P* < 0.05. Notably, the model outperformed the best-performing orthopaedic surgeon in all evaluated parameters.

## Discussion

Pediatric hip and periarticular infections present with various manifestations, including septic arthritis, adjacent osteomyelitis, and intramuscular abscesses. There is a widespread consensus that early and thorough debridement is necessary for joint abscesses, subperiosteal abscesses, and intramuscular abscesses [1–10]. The aim of this study was to construct an AI model to assist in surgical planning for orthopaedic surgeons. The results showed that the Mask R-CNN model was reliable for identifying osteomyelitis and abscess in pediatric hip and periarticular infections, offering a more convenient and rapid option.

Deep learning, a sophisticated neural network resembling the human brain, has demonstrated the ability to tackle intricate problems that were previously challenging for low-level AI. In the medical field, deep learning has exhibited proficiency in interpreting two-dimensional images, comparable to that of a human expert [27]. Convolutional Neural Networks (CNNs) have swiftly become a crucial method for analyzing medical images, particularly in image recognition and visual learning tasks. Numerous studies across various medical fields, including X-ray, ultrasound, CT, MRI, microscopy, and endoscopy, have reported promising results in diagnosis and classification using CNNs [28–33]. ResNet, as exemplified by He et al. [34], not only address the issue of gradient vanishing by allowing gradients to pass through shortcut paths but also enable the learning of identity functions, ensuring that higher-level performance matches or surpasses the underlying layer. Moreover, Faster R-CNN, introduced in 2015, is recognized for its powerful processing speed in target detection. However, Mask R-CNN is an improved network model based on the Faster R-CNN framework. In addition to the class labels and bounding box offsets produced by Faster R-CNN, Mask R-CNN adds a segmentation mask corresponding to the output of the third branch to the input samples, by proposing a binary mask for each ROI.

Detecting target lesions in medical images and accurately segmenting them pose significant challenges. These two tasks are often considered as two independent processes, and using a multi-task framework may lead to false edges and systemic errors. Mask R-CNN introduces the mask branch to maintain a clear spatial layout of objects, which not only enhances lesion segmentation accuracy but also requires fewer parameters, resulting in minimal computational overhead. Specifically, in the process of proposing candidate box object, a mask branch parallel to the classification and bounding box regression branches is added. The ROI Align layer, implemented through bilinear interpolation, replaces the ROI Pooling layer, computing precise values of input features at four regularly sampled positions within each RoI bin. This addresses pixel-level misalignment caused by spatial quantization. In addition, the ResNet-101 and the Feature Pyramid Network (FPN) were used as the backbone network, making training simple and flexible, leading to improved accuracy and speed. In this study, Mask R-CNN was used to precisely remove unnecessary information from the original images and segment the complete infected regions, including osteomyelitisand abscess. This approach provided effective data augmentation, enabling the model to focus on critical target regions and improve the overall performance. Additionally, the model could detect, classify and display risk probabilities in real-time. This can assist doctors in visually assessing the risk level of infected areas directly and identifying the core of lesions.

**Fig. 8:**
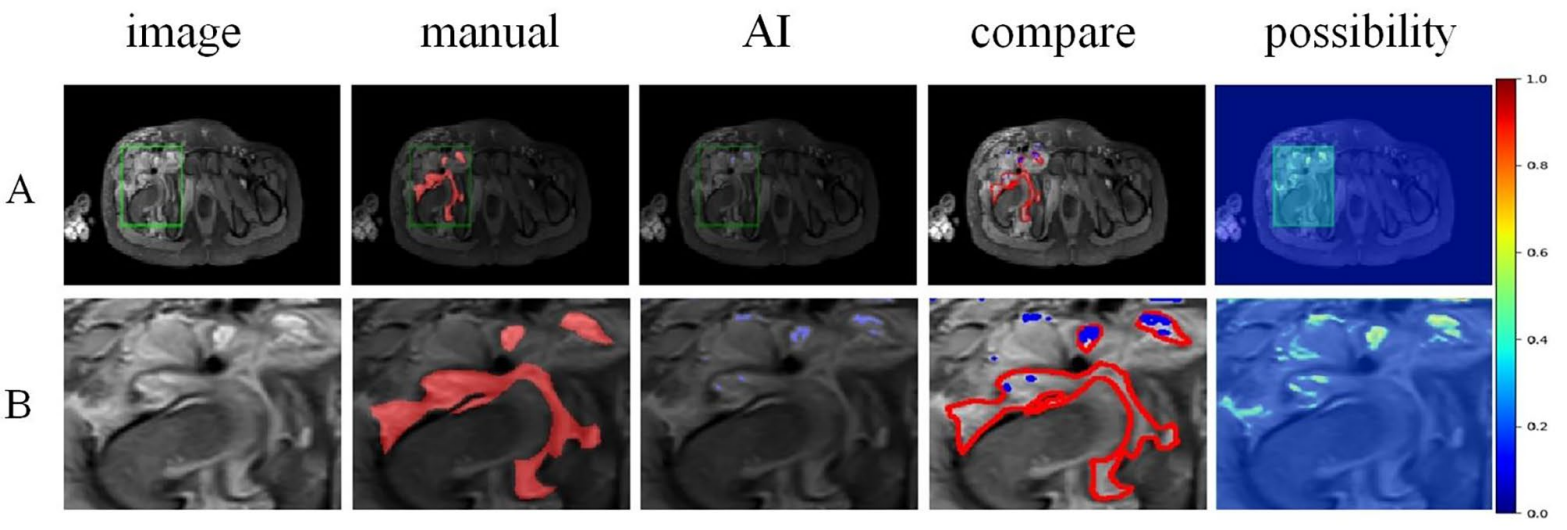
For chronic abscess, STIR images showed heterogeneous abnormal signals and discrepancies between specialists and AI diagnosis occured, but AI was able to show residual high signal areas of fluid through colour risk prediction (**Figure A** and **B**)

The model was more sensitive to identify bone marrow edema and even compensated for the omission of expert judgement in some images. Moreover, as the progression of disease and conservative treatment, the manifestations of infections could change correspondingly, especially when abscess enters the subacute or chronic stage, leading to varying degrees of absorption and fibrosis. This was reflected in changes in STIR images(Figure 8), which made accurate labeling challenging. However, AI learning was based on the imaging characteristics of the target lesion, which was more objective and precise, and the colour risk prediction showed that the results of AI were more accurate compared to the subjective judgement of the specialists. Future work will involve adding more training datasets to the network to improve the accuracy and reliability of the model’s classification and segmentation results. This study has the following limitations. Firstly, a subset of the included MRI images (64 cases) were re-exmaine images taken after conservative treatment without improvement within 3–5 days. The differences in the inflammatory manifestations may have influenced the effectiveness of the model’s training. Secondly, due to the wide range of soft tissue infections involving bone joints, the associated information on MRI images is complex. However, this model was limited to learn and recognize the content with distinct imaging characteristics, such as osteomyelitis and abscess, and could not assess the overall extent of the infection. In conclusion, this study demonstrated the feasibility of an AI-assisted MRI model based on Mask R-CNN for identifying surgical target areas in pediatric hip and periarticularinfections. It can assist unexperienced physicians in pre-treatment assessments, helping them avoid oversights.

## Data Availability

The datasets used and/or analyzed during the current study are available from the corresponding author on a reasonable request.
